# Case report of concurrent ulcerative colitis and bilateral breast cancer: a literature review of the bidirectional association between autoimmune diseases and breast cancer

**DOI:** 10.3389/fonc.2025.1495731

**Published:** 2025-06-11

**Authors:** Xiao Qi, Miao Zhang, Jinhong Zhu, Huaqing Wang

**Affiliations:** ^1^ Department of Oncology, Tianjin Union Medical Center, The First Affiliated Hospital of Nankai University, Tianjin, China; ^2^ Tianjin Cancer Institute of Integrative Traditional Chinese and Western Medicine, Tianjin, China; ^3^ The Institute of Translational Medicine, Tianjin Union Medical Center, The First Affiliated Hospital of Nankai University, Tianjin, China; ^4^ Nankai University School of Medicine, Tianjin, China

**Keywords:** ulcerative colitis, bilateral breast cancer, adjuvant chemotherapy, target therapy, autoimmune disease

## Abstract

A 58-year-old female patient with bilateral breast cancer developed unexpected hematochezia at a frequency of approximately 10 episodes per day following adjuvant chemotherapy, with the emergency endoscopy reporting superficial ulcers throughout the entire colon, suggesting a diagnosis of ulcerative colitis (UC). Given the absence of preexisting autoimmune history or detectable autoantibodies, we supposed that the onset of UC was closely related to the chemotherapy. The complex bidirectional relationship between autoimmune rheumatic diseases and cancer continues to be elucidated. Variations in autoimmune disease type, duration, and specific clinical/laboratory features may modulate cancer risk, either increasing or decreasing susceptibility to certain malignancies. These associations could potentially inform type-specific cancer screening strategies. Furthermore, the widespread use of immune checkpoint inhibitors across multiple tumor types, along with their associated inflammatory syndromes, has significant implications for the development and management of autoimmune rheumatic diseases. Herein, we report this case, which could be one of the few bilateral breast cancer cases to be reported with ulcerative colitis, and conducted a literature review of the bidirectional association of breast cancer and autoimmune diseases.

## Introduction

Ulcerative colitis (UC), one of the two primary forms of inflammatory bowel disease (IBD), is characterized by mucosal inflammation extending continuously from the rectum to the proximal colon (1). While the exact etiology of IBD remains incompletely understood, current evidence suggests a multifactorial pathogenesis involving molecular and cellular mechanisms, microbial factors, microbiome interactions, genetic predisposition, and immune dysregulation (2). Similar to other chronic inflammatory conditions, UC is associated with an elevated risk of malignancy. Notably, colitis-associated cancer (CAC)—colorectal cancer being the most prevalent—demonstrates risk stratification based on factors including age at diagnosis, disease duration, and disease severity (3). Given the elevated cancer risk associated with autoimmune diseases (AIDs), there has been growing attention on the long-term management of patients with pre-existing AIDs (4). Recent studies have yielded divergent findings regarding the cancer risks of different AIDs, with some conditions, such as rheumatoid arthritis (RA), potentially conferring a reduced risk rather than an increased one (5). Using breast cancer as an example, recent research has revealed that the impact of autoimmune disorders on breast cancer development extends far beyond the initially recognized association with thyroid disease, particularly the involvement of both anti-thyroid peroxidase (anti-TPO) and anti-thyroglobulin (anti-TG) antibodies (6, 7). Furthermore, mounting evidence confirms a bidirectional interplay between AIDs and cancer, wherein certain malignancies may significantly alter susceptibility to subsequent autoimmune conditions. This relationship is exemplified by a nationwide population-based cohort study (Chen et al.), which revealed that breast cancer patients showed markedly reduced incidence rates of systemic lupus erythematosus (SLE), RA, and Sjögren’s syndrome (SS) compared to matched controls (8, 9).

## Case presentation

**Figure 1:**
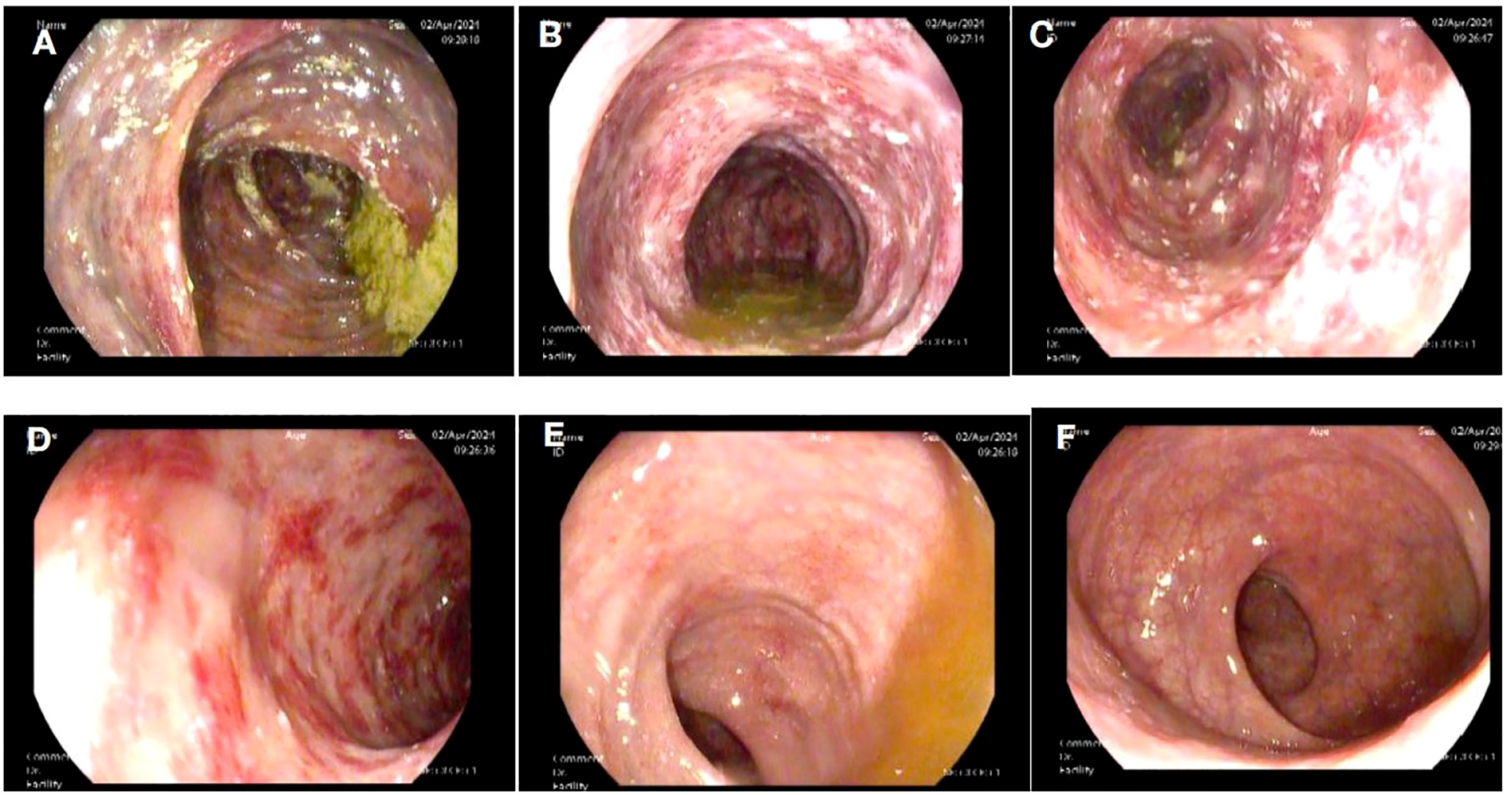
Superficial ulcers involving the whole colon. Superficial ulcers in the ascending colon **(A)**, transverse colon **(B)**, descending colon **(C)**, and sigmoid colon **(D)**. No obvious ulcers in the rectosigmoid junction **(E)** and rectum **(F)**.

**Figure 2:**
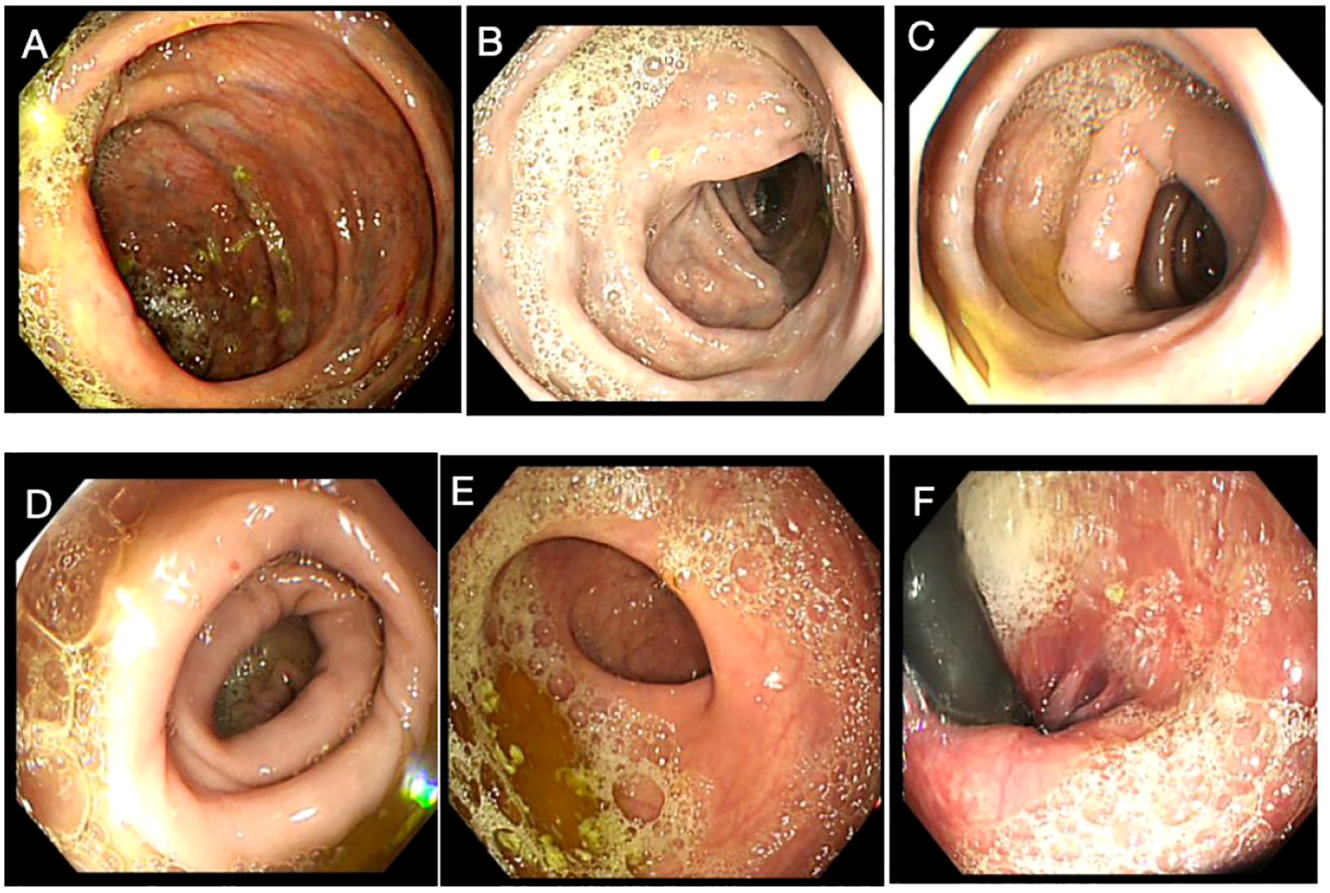
Endoscopic findings upon remission of the colitis. No obvious ulcers were detected in the ascending colon **(A)**, transverse colon **(B)**, descending colon **(C)**, sigmoid colon **(D)**, rectosigmoid junction **(E)**, and rectum **(F)**.

**Figure 3:**
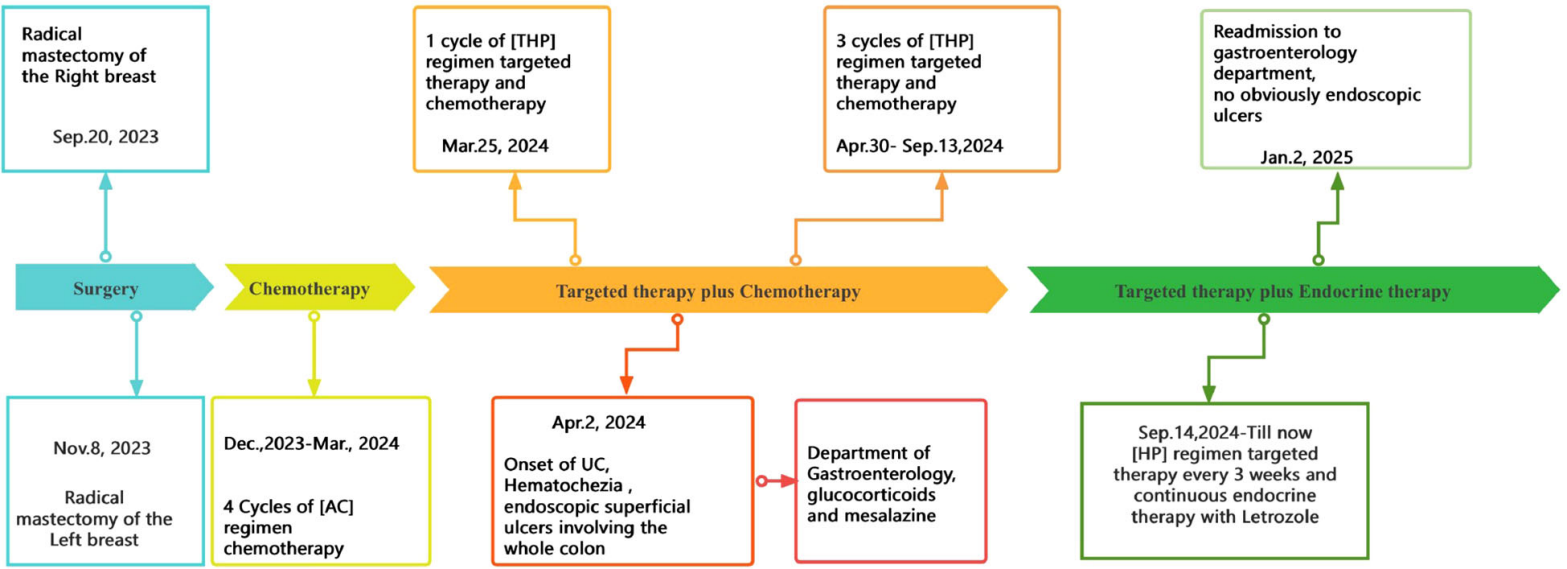
Complete timeline summarizing the main events of the patient’s treatment.

A 58-year-old female patient underwent sequential radical mastectomies, first on the right breast (20 September 2023) and then followed by the left breast (8 November 2023). Histopathological analysis revealed the following: Right breast: invasive ductal carcinoma (Estrogen receptor (ER) >90%, Progesterone receptor (PR) 60%, Human epidermal growth factor receptor 2 (Her2) 1+, Ki-67 10-20%), pathological stage pT2N0Mx; Left breast: predominantly ductal carcinoma *in situ* with focal invasive ductal carcinoma (ER <1%, PR <1%, HER2 3+, Ki-67 40%), pathological stage pT1bN0Mx (**Table 1**). The patient initiated the AC-THP adjuvant chemotherapy regimen, completing four cycles (cyclophosphamide 0.8g d1 + doxorubicin liposome 50mg d1, Q3W). During the fifth cycle (trastuzumab 560mg d0 + pertuzumab 840mg d0 + albumin-bound paclitaxel 400mg d1), she developed acute hematochezia (approximately 10 episodes per day). Emergency endoscopy demonstrated diffuse superficial colonic ulcers (**Figure 1**), though a biopsy was refused by the patient due to bleeding concerns. Given the result of negative stool cultures and C. difficile toxin assays, the absence of serum autoimmune antibodies (**Table 2**), and the clinical improvement following glucocorticoid and mesalazine therapy, combined with the endoscopic findings, the patient was diagnosed with UC. Follow-up endoscopy (January 2, 2025) showed complete ulcer resolution (**Figure 2**). The complete treatment timeline summarizing the main events is shown in **Figure 3**.

**Table 1:** Details in postoperative histo-pathological information.

Pathological description and immunohistochemistry	Right breast	Left breast
Pathological type	Invasive ductal carcinoma	ductal carcinoma in situ, with some areas of invasive ductal carcinoma
Histological grade	grade II	grade II-III
Lymphocyte infiltration	<5%	30%
Vascular tumor emboli	+	–
ER	>90%	<1%
PR	60%	<1%
Her-2	+	+++
Ki-67	10%-20%	40%
P53	5%	80%
EGFR	<1%	10%
CK5/6	<1%	<1%
AR	>90%	70%
Lymph nodes	0/19	0/11
Surgical margin	Negative	Negative
Pathological staging	pT2N0Mx	pT1bN0Mx
Remarks		Paget's disease of the nipple

ER, Estrogen receptor; PR, Progesterone receptor; Her2, Human epidermal growth factor receptor 2; EGFR, Epidermal growth factor receptor; AR, Androgen receptor.

**Table 2:** Indicators of autoimmune antibodies.

Antibodies	Abbreviation	Index	Normal range
Thyroid antibodies			
Thyroid globulin antibodies	TG	(-)	(-)
Thyroid peroxidase antibodies	TPO	(-)	(-)
ANCA			
Anti-neutrophil cytoplasmic antibodies	ANCA	(-)	(-)
Anti-nuclear antibodies (ANAs)			
Anti-nuclear ribonucleoprotein/Smith antibodies	nRNP/Sm	(-)	(-)
Anti-Smith antibodies	Sm	(-)	(-)
Anti-Sjögren’s syndrome-A antibodies	SS-A	(-)	(-)
Anti-Sjögren’s syndrome-B antibodies	SS-B	(-)	(-)
Anti-Ro-52 antibodies	KRO	(-)	(-)
Anti-Jo-1 antibodies	Jo-1	(-)	(-)
Anti-Scleroderma-70 antibodies	scl-70	(-)	(-)
Anti-PM-Scl antibody	PM-scl	(-)	(-)
Anti-centromere antibodies	CENPB	(-)	(-)
Anti-proliferating cell nuclear antigen antibodies	PCNA	(-)	(-)
Anti-double-stranded deoxyribonucleic acid antibodies	dsDNA	(-)	(-)
Anti-nucleosome antibodies	Nucleosome	(-)	(-)
Anti-histone antibodies	Histones	(-)	(-)
Anti-ribosomal P protein antibodies	ARPA	(-)	(-)

## Discussion

Bilateral breast cancer represents a distinct and relatively uncommon form of malignant breast neoplasia, characterized by independent primary tumors originating from the ductal and/or lobular epithelium in both breasts, either synchronously or metachronously. Since Kilgore’s initial description in 1921 (10), reported cases have progressively increased, with synchronous bilateral breast cancer (SBBC) constituting ≤5% of all breast malignancies (11). UC, an idiopathic chronic inflammatory disorder of the colonic mucosa (with potential pancolonic involvement), typically manifests with relapsing-remitting hematochezia (12). We herein present a rare case of synchronous bilateral breast cancer complicated by UC. The mechanisms underlying CAC involve multiple molecular pathways that may include genetic mutations, such as TP53 gene alterations; inflammatory signaling, such as TLR4-MyD88-dependent NF-κB activation, which perpetuates a pro-inflammatory microenvironment; the prostaglandin pathway, such as COX-2-mediated PGE2 production, promoting angiogenesis and immune modulation via PI3K signaling; metabolic reprogramming, such as PI3K/AKT activation by cytokines that facilitates GLUT4 translocation, enhancing TH2 and TH17 lymphocyte metabolism; genetic regulation, such as modifications in key regulatory genes (IL23R and rs10889677 [c.*309C>A]) of the PI3K/AKT signaling pathway. These molecular alterations interact with immune components (macrophages, neutrophils, and T-regulatory cells) and cellular processes (autophagy), ultimately driving CAC development (13).

Similarities in the pathologies of autoimmune diseases and cancers can be traced back at least 30 years. Autoimmune diseases result from the breakdown of peripheral immune tolerance, specifically, the disruption of the delicate balance between immune tolerance and immune activation. This equilibrium is normally maintained by specialized cell subsets, such as regulatory T cells (Tregs) and B cells (Bregs), macrophages, fibroblasts, and myeloid-derived suppressor cells (MDSCs) (14). When this balance is disturbed, it leads to pathological immune overactivation. While the recruitment of tolerogenic immune cell subsets and evasion of immune surveillance are recognized as the hallmarks of cancer, often termed “cancer tolerance”, malignant cells achieve this through multiple mechanisms. These include the expression of immune checkpoint proteins, impaired antigen presentation, epithelial-to-mesenchymal transition (EMT), and dysregulated RNA editing. A very important part of this complex network is the microbiome, as both the microbiome and its metabolites can exert pro-tumor function, and a gut microbiota imbalance can cause gastrointestinal cancers or IBD (15). Inflammatory cytokines and growth factors mediate cell proliferation, and proteinases, especially the collagenase, matrix metalloproteinase-1 (MMP-1), contribute to disease progression by remodeling the extracellular matrix and modulating the microenvironment (16). Chronic inflammation caused by autoimmune diseases or the anti-rheumatic therapies and oncogenic virus infections, such as human papillomavirus, hepatitis B and C viruses, and Epstein–Barr virus, which are difficult to treat, may contribute to the development and progression of cancer (8). Autoimmuity-induced chronic inflammation and tissue damage may produce inflammatory mediators, such as TNF-α, interleukin (IL)-6, tumor growth factor (TGF)-β, and IL-10, leading to the failure of Treg-mediated immune tolerance and stimulating the development of malignant tumors through various mechanisms such as DNA damage, inactivation of tumor-suppressor genes, triggering of angiogenesis, and enhancing invasiveness (17).

Conversely, anti-tumor immune responses may cross-react with self-tissues, leading to the development of autoimmunity (6). Previous studies indicate that patients with pre-existing autoimmune diseases often exhibit shortened survival, primarily attributable to the direct or indirect immune-related adverse events (irAEs) induced by anti-tumor therapies such as chemotherapy, radiation therapy, and, notably, the accumulated application of immune checkpoint inhibitor in the management of advanced tumors (18), leading to the flare-up or onset of autoimmune diseases (8, 19, 20). Numerous chemotherapeutic agents have been implicated in the development of autoimmune-like manifestations, especially bleomycin, gemcitabine, carboplatin, and paclitaxel have been associated with scleroderma-like syndromes and Raynaud’s phenomenon (21). Aromatase inhibitors (AIs), a mainstay of hormone receptor-positive breast cancer treatment, frequently induce arthralgias (22). Radiotherapy targeting the head and neck regions can induce xerostomia that clinically resembles primary Sjögren’s syndrome. Emerging evidence suggests that approximately 50% of breast cancer patients may develop radiation-induced cutaneous fibrosis at treatment sites, though current studies remain limited by small cohort sizes and methodological constraints (23). In patients with SLE, low levels of transforming growth factor-beta 1 (TGF-β1) correlate with increased disease severity. Therefore, radiotherapy-induced TGF-β release may have therapeutic potential in SLE by compensating for this deficiency (24). ICIs induce uncontrolled T-cell activation, resulting in systemic inflammatory syndromes that may involve multiple organ systems. These irAEs can manifest as autoimmune-like pathologies affecting the skin, endocrine, gastrointestinal, cardiovascular, pulmonary, and neurological systems (25, 26). The patient in this case report received multiple chemotherapy, targeted, and endocrine therapies. While treatment-induced colitis has not been previously reported with these specific regimens, the potential for immune-related adverse events, including autoimmune-like manifestations such as colitis, cannot be ruled out.

### Autoimmune thyroid diseases

The association between breast cancer and thyroid autoimmunity has attracted sustained research interest owing to the shared biological characteristics of thyroid and mammary gland tissues. A previous meta-analysis (27) demonstrated an elevated breast cancer risk among patients with autoimmune thyroiditis, particularly in those with anti-thyroid peroxidase (anti-TPO) and anti-thyroglobulin (anti-TG) antibodies. This association may stem from shared iodine-concentrating capacity via the sodium/iodide symporter (NIS) in both epithelial tissues or the abundance of TSH receptor-rich adipose tissue in breast parenchyma (28). While NIS protein expression is too limited to serve as a primary autoantigen, current evidence highlights the following two key antigenic links: thyroid peroxidase and its mammary homolog lactoperoxidase (both expressed in breast tissue) (29) and thyroid hormone receptor α 2 (TRα2), which modulates breast cancer signaling pathways. Notably, TRα2 overexpression correlates with improved survival outcomes in patients with breast cancer and is particularly upregulated in BRCA1-associated breast cancers, serving as a positive prognostic marker for both 5-year and overall survival. Additional mechanistic insights include the presence of estrogen receptors in abnormal thyroid tissue, potentially explaining thyroid dysfunction in breast cancer patients and shared endocrine stimuli affecting both organs (30, 31). These findings suggest diagnostic and therapeutic opportunities through targeting thyroid-breast antigenic connections (6).

### Rheumatoid arthritis

RA has been associated with an increased risk of hematological and solid malignancies for many years, even more common during treatment with disease-modifying anti-rheumatic drugs (DMARDs) and biotherapy (32). However, in recent years, multiple studies have demonstrated that RA does not uniformly elevate cancer risk, but rather exhibits tumor-specific and sex-dependent variations in malignancy incidence. Patients with RA have an increased risk of non-Hodgkin lymphoma, but a lower risk of gastric cancer (33). Men have a reduced risk of rectal and renal malignancies, while women have a reduced risk of gastric and rectal cancers, and a reduced risk of hepatic malignancies is found in both men and women (34). Regarding hematological tumors, the risk of lymphoma was significantly higher in RA patients of both sexes, but the incidence of leukemia was significantly lower in women (35).

Epidemiological studies demonstrate a bidirectional reduction in disease risk between RA and breast cancer. Women with RA show decreased breast cancer incidence, while breast cancer survivors exhibit similarly reduced RA risk (5, 36). Notably, adjuvant antihormonal therapies (tamoxifen or aromatase inhibitors) do not increase RA risk compared to other breast cancer treatments, a finding replicated in Chinese populations (7). The MMP-1 system reveals important pathophysiological connections between these conditions. Initially characterized for its collagenolytic activity, MMP-1 now demonstrates pleiotropic functions through interactions with Chemokine receptor CXCR-4 and protease-activated receptor-1 (PAR-1). These interactions establish critical signaling networks. The MMP-1/CXCR4 axis modulates activated fibroblast behavior in both RA and cancer. The MMP-1/PAR-1 autocrine/paracrine loop amplifies extracellular matrix remodeling and cellular responses. Emerging therapies targeting MMP-1 and associated G protein-coupled receptors highlight shared pathogenic mechanisms between autoimmunity and oncogenesis (10). While more specific than previous agents, these therapies underscore the fundamental biological parallels between RA and certain malignancies.

### Systemic lupus erythematosus

The association between SLE and breast cancer remains controversial. While early studies suggested a protective effect of SLE against breast cancer, more recent investigations have yielded conflicting results. A Brazilian cohort study (n=100) reported elevated breast and cervical cancer incidence in patients with SLE vs. the general population (37). Large international studies demonstrated reduced breast cancer risk in patients with SLE (38, 39). Age-adjusted analyses show comparable or slightly increased breast cancer risk in patients with SLE vs. controls (40). A multicenter international study (n=16,409) found no SLE-specific factors explaining cancer risk variations (41). Longitudinal data indicate that age is the primary breast cancer risk factor in patients with SLE (41). A Mendelian randomization analysis revealed population-specific effects, with a significant risk reduction in East Asian cohorts (OR 0.85, 95% CI 0.78-0.93) but no association in European populations (42). A five-gene SLE prognostic signature (RACGAP1, HMMR, TTK, TOP2A, and KIF15) effectively stratified breast cancer survival risk (43). These contradictory findings highlight the need for more large prospective cohort studies, population-specific risk assessments, and mechanistic investigations into SLE-related immunomodulatory effects.

### Other autoimmune diseases

A study based on a Spanish population showed an increased overall cancer risk in patients with systemic sclerosis (SSc) compared with the general population. A high risk in lung, breast cancer, and hematological malignancies was also reported, with the presence of anticentromere antibodies an independent favorable factor for decreasing cancer risk (44). A similar outcome was reported in a large-scale cohort study conducted in China (4). For primary Sjögren’s syndrome (pSS), there could be geographical differences in its association with breast cancer risk; patients with pSS in European countries exhibited a lower risk of breast cancer, different from Asia and Argentina (44). Dedousis et al. conducted a survival analysis that found that there was a higher prevalence of rheumatoid arthritis, Crohn’s disease, ulcerative colitis, and systemic lupus erythematosus in patients with breast cancer compared to age-matched cohorts in the general population. The presence of an autoimmune diagnosis was associated with a lower overall survival (OS) in stages I–III breast cancer and improved OS in patients with stage IV disease, suggesting that anti-tumor immunity plays an important role in advanced breast cancer and could potentially be exploited to improve the effectiveness of immunotherapy (45). High breast cancer risk was also found in type 1 diabetes mellitus (T1DM) and psoriasis (Pso) (5, 46), whereas breast cancer patients with multiple sclerosis suffered a modest progression during breast cancer treatment (47). It is generally accepted that during the 3 to 5 years before and after the diagnosis, patients with inflammatory myopathies have the highest risk of developing certain types of cancers, with adenocarcinoma being the most common histological type, among which the breasts could be a susceptible site (48). In autoimmune retinopathy, anti-TULP1 AAbs disrupt protein translocation to the outer segments and are involved in photoreceptor degeneration in a manner similar to the degeneration induced by mutations in the TULP1 gene. The detection of anti-TULP1 AAbs in patients with breast cancer suggests that anti-TULP1 AAbs have the potential to be a biomarker for cancer-associated autoimmune retinopathy (49).

## Conclusion

Breast cancer and autoimmune diseases represent two prevalent conditions that threaten women’s health, with growing evidence suggesting their complex bidirectional relationship. This case highlights the co-occurrence of breast cancer and UC, offering several important insights. While colorectal cancer remains the predominant malignancy associated with UC, this case suggests the potential oncogenic effects of UC beyond the colon. The patient’s diverse therapeutic regimens (chemotherapy, targeted agents, and endocrine therapy) may have contributed to UC development, consistent with established evidence linking anticancer treatments and autoimmune-like manifestations. However, there is also a limitation as the exact causal relationship between breast cancer and UC in this case remains undetermined. This also calls for clinical vigilance regarding autoimmune manifestations in cancer patients and the requirement for large-cohort studies to elucidate the disease-disease interactions and treatment-related autoimmune effects to provide guidelines for oncologists, immunologists, and gastroenterologists to optimize care for patients with overlapping cancer and autoimmune diseases.

## Data Availability

The raw data supporting the conclusions of this article will be made available by the authors, without undue reservation.
